# Supplementary material for: Advancing 7T perfusion imaging by pulsed arterial spin labeling: Using a parallel transmit coil for enhanced labeling robustness and temporal SNR
Source: PLoS One. 2024 Aug 26;19(8):e0309204. doi: 10.1371/journal.pone.0309204 (PMC11346640; doi:10.1371/journal.pone.0309204)
Supplement: S1 File — This supplementary file presents detailed results from the linear regression analysis, systematically summarized in S1-S4 Tables. It also features S1, S2 Figs, which illustrate B1+ maps and individual perfusion maps, providing more details for both coil configurations. (PDF) [file pone.0309204.s001.pdf]

Supplementary material

Linear Regression Analysis

We ran the linear regression on right and left Gray Matter (GM) masks separately.

S1 Table: Linear regression for 2Tx Right Gray Matter

Coefficient	Estimate	Std..Error	t.value	p.value
intercept	0.34887	0.23211	1.503	0.14928
slope	0.07337	0.02489	2.948	0.00825

Residual standard error: 0.2793 on 19 degrees of freedom
Multiple R-squared: 0.3139, Adjusted R-squared: 0.2778
F-statistic: 8.693 on 1 and 19 DF, p-value: 0.008252

S2 Table: Linear regression for pTx Right Gray Matter

Coefficient	Estimate	Std..Error	t.value	p.value
intercept	0.34702	0.27180	1.277	0.2171
slope	0.05263	0.02914	1.806	0.0868

Residual standard error: 0.3271 on 19 degrees of freedom
Multiple R-squared: 0.1465, Adjusted R-squared: 0.1016
F-statistic: 3.262 on 1 and 19 DF, p-value: 0.08675

S3 Table: Linear regression for 2Tx Left Gray Matter

Coefficient	Estimate	Std..Error	t.value	p.value
intercept	0.26651	0.12776	2.086	0.0507
slope	0.03731	0.01370	2.723	0.0868

Residual standard error: 0.1538 on 19 degrees of freedom
Multiple R-squared: 0.2808, Adjusted R-squared: 0.2429
F-statistic: 7.417 on 1 and 19 DF, p-value: 0.01349

S4 Table: Linear regression for pTx Left Gray Matter

Coefficient	Estimate	Std..Error	t.value	p.value
intercept	0.22356	0.16585	1.348	0.1935
slope	0.04245	0.01778	2.388	0.0275

Residual standard error: 0.1996 on 19 degrees of freedom
Multiple R-squared: 0.2308, Adjusted R-squared: 0.1903
F-statistic: 5.7 on 1 and 19 DF, p-value: 0.02751

Supplementary Figures

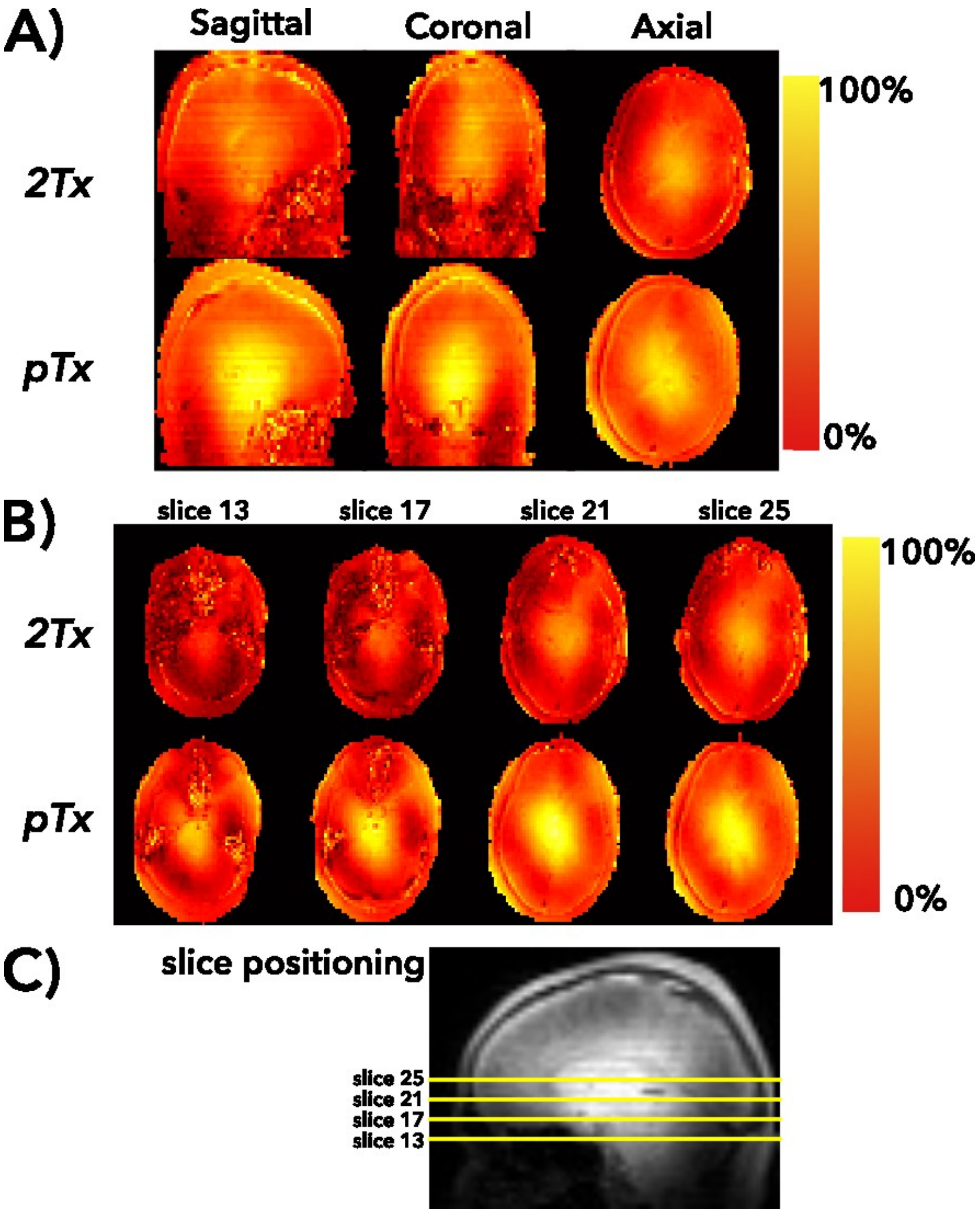

S1 Fig: Representative $B1^+$ maps (DREAM B1) from an individual participant. Panel A depicts the $B1^+$ maps in sagittal, coronal and axial planes. Panel B shows the location of four slices, corresponding to the typical location of the labeling plane. Noticeably higher B1 values are observed in the pTx compared to the 2Tx B1 map. Panel C displays the location of the four slices.

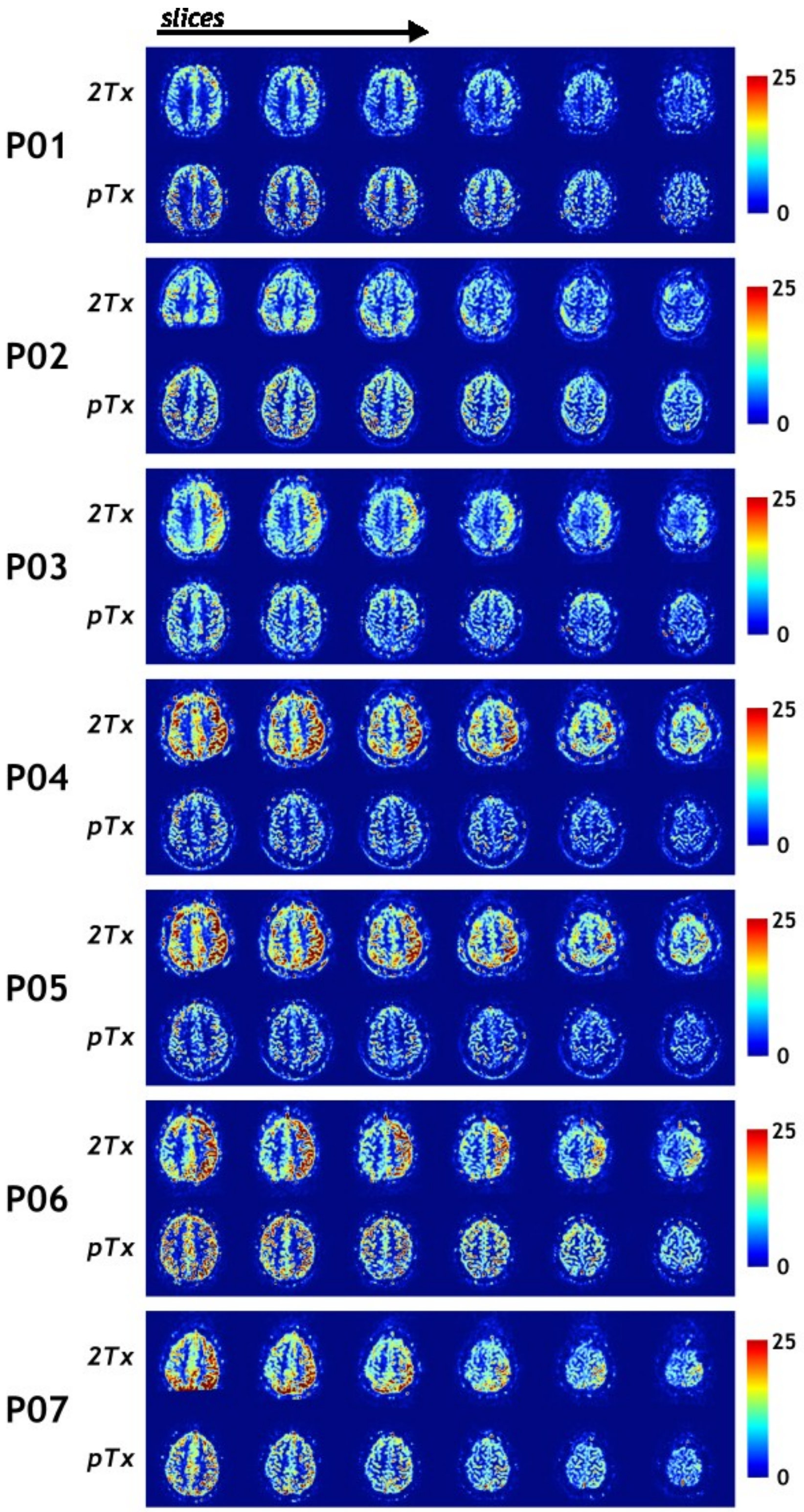
slices
P01
2Tx
pTx
P02
2Tx
pTx
P03
2Tx
pTx
P04
2Tx
pTx
P05
2Tx
pTx
P06
2Tx
pTx
P07
2Tx
pTx
25
0

S2 Fig: Six slices (from 6-11) of perfusion-weighted ASL from all seven participants highlighting differences between the 2Tx and pTx systems. The pTx system generally shows a more homogeneous signal (e.g. fewer right-left differences) than the 2Tx. The perfusion maps are from the 6 cm slab inversion thickness.
