# Supplementary material for: Advancing 7T perfusion imaging by pulsed arterial spin labeling: Using a parallel transmit coil for enhanced labeling robustness and temporal SNR
Source: PLoS One. 2024 Aug 26;19(8):e0309204. doi: 10.1371/journal.pone.0309204 (PMC11346640; doi:10.1371/journal.pone.0309204)
Supplement: Attachment — Submitted filename: response_to_reviewers_v3.docx [file pone.0309204.s002.docx]

**Journal requirements**

When submitting your revision, we need you to address these additional requirements.

1. Please ensure that your manuscript meets PLOS ONE's style requirements, including those for file naming. The PLOS ONE style templates can be found at

<https://journals.plos.org/plosone/s/file?id=wjVg/PLOSOne_formatting_sample_main_body.pdf> and 
<https://journals.plos.org/plosone/s/file?id=ba62/PLOSOne_formatting_sample_title_authors_affiliations.pdf>

Response: We have now formatted the manuscript accordingly.

2. Please note that PLOS ONE has specific guidelines on code sharing for submissions in which author-generated code underpins the findings in the manuscript. In these cases, all author-generated code must be made available without restrictions upon publication of the work.

Please review our guidelines at <https://journals.plos.org/plosone/s/materials-and-software-sharing#loc-sharing-code> and ensure that your code is shared in a way that follows best practice and facilitates reproducibility and reuse.

Response: All in-house scripts developed for this study can be accessed via the provided GitHub page (https://github.com/icaroafoliveira/ASL_7T_pTx_scripts).

3. We note that the grant information you provided in the ‘Funding Information’ and ‘Financial Disclosure’ sections do not match.

When you resubmit, please ensure that you provide the correct grant numbers for the awards you received for your study in the ‘Funding Information’ section.

Response: Thank you for pointing this out. We fixed that in the rebuttal submission.

4. Thank you for stating the following financial disclosure:

"This work was supported by a Royal Netherlands Academy for Arts and Sciences (KNAW) research grant NWO (Nederlandse Organisatie voor Wetenschappelijk Onderzoek), Number: TTW VI.Vidi.198.016 to W.Z."

Please state what role the funders took in the study.  If the funders had no role, please state: "The funders had no role in study design, data collection and analysis, decision to publish, or preparation of the manuscript." If this statement is not correct you must amend it as needed.

Please include this amended Role of Funder statement in your cover letter; we will change the online submission form on your behalf.

Response: Additional information about the financial disclosure was added to the cover letter. "The funders had no role in study design, data collection and analysis, decision to publish, or preparation of the manuscript."

5. We note that you have indicated that there are restrictions to data sharing for this study. For studies involving human research participant data or other sensitive data, we encourage authors to share de-identified or anonymized data. However, when data cannot be publicly shared for ethical reasons, we allow authors to make their data sets available upon request. For information on unacceptable data access restrictions, please see <http://journals.plos.org/plosone/s/data-availability#loc-unacceptable-data-access-restrictions>.

Before we proceed with your manuscript, please address the following prompts:

a) If there are ethical or legal restrictions on sharing a de-identified data set, please explain them in detail (e.g., data contain potentially identifying or sensitive patient information, data are owned by a third-party organization, etc.) and who has imposed them (e.g., a Research Ethics Committee or Institutional Review Board, etc.). Please also provide contact information for a data access committee, ethics committee, or other institutional body to which data requests may be sent.

b) If there are no restrictions, please upload the minimal anonymized data set necessary to replicate your study findings to a stable, public repository and provide us with the relevant URLs, DOIs, or accession numbers. Please see <http://www.bmj.com/content/340/bmj.c181.long> for guidelines on how to de-identify and prepare clinical data for publication. For a list of recommended repositories, please see <https://journals.plos.org/plosone/s/recommended-repositories>. You also have the option of uploading the data as Supporting Information files, but we would recommend depositing data directly to a data repository if possible.

Please update your Data Availability statement in the submission form accordingly.

Editor comments:

Regarding data availability, I don't share the authors view. GDPR allows publication of raw data if they are de-identified, that is if any connection between data and individual are broken. If MR images can't be associated to a person, then they are not perosnal data and are not regulated by GDPR. Of course, any personal feature (including faces in anatomical data) must be deleted.

I understand the concerns (I am an UE citizen and work in EU), but the open data principles must be adhered to. I suggest to check with the local privacy officer.

**Response:**

Unfortunately, this is not our view, but a legal restriction explained to us first by the privacy officer of the Royal Academy of Arts and Sciences (KNAW). To clarify this further, we have contacted a legal advisor who has drawn up an advice for the KNAW on (f)MRI scans and the AVG (the Dutch name for GDPR). They state clearly that brain scans are unique, like fingerprints and should be considered personal information as this unique information can be used for identification, even after defacing. All future data sharing should be covered by a data sharing agreement between recipient and the KNAW. Requests can be sent to [w.vanderzwaag@spinozacentre.nl](mailto:w.vanderzwaag@spinozacentre.nl).

**Response to reviewers**

**Reviewer #1:** In this work, the authors compared the effects of different transmit coils (two-channel v.s. 8-channel) on multi-slice cerebral pulsed arterial spin labeling (pASL) at 7T. The comparison was applied to both baseline with three different inversion slab thicknesses and functional ASL of the hand region in the motor cortex with a finger-tapping functional task. The author found lower coefficient of variance (CoV) between the perfusion signal and the inversion slab thickness using 8-channel transmit than two-channel, which represents a higher robustness. The author also demonstrated higher temporal SNR and comparable capabilities for functional ASL using the 8-channel transmit coil.
The experiments in this work were well-designed. The novelty of this manuscript is moderate since pASL has not been a preferred method for brain perfusion mapping in recent years. This manuscript is well written with a clear structure and proper language. Here are some minor tips:

We thank the reviewer for the time taken to review the present manuscript. We appreciate all positive comments and suggestions. In the context of moderate field strengths (1.5 and 3T), we agree with the reviewer that pulsed ASL has not been a preferred method for brain perfusion mapping. However, as the use of the preferred pCASL method is challenging at 7T, pulsed ASL remains a popular method at high-field MRI [1] as well for functional ASL experiments in which the shorter temporal footprint of PASL is an advantage over pCASL. We therefore believe that this method is still relevant for the ultra-high field community, especially in the context of functional MRI (fMRI). Please find the detailed answers to the reviewer’s comments below.

1. The title ‘Advancing 7T perfusion imaging by Arterial Spin Labeling: Using parallel transmit for enhanced labeling efficiency and image quality’. It is recommended to specify ‘Pulsed’ Arterial Spin Labeling. Also, ‘enhanced labeling efficiency and image quality’ is too general since there are no results involving labeling efficiency as a metric. It is better to specify ‘higher robustness’ and ‘higher temporal SNR’. Besides, the running title does not even mention ‘parallel transmit’, which is the key of this manuscript.

Response: We agree with the reviewer about the generalisability of our current manuscript title. We changed the title to “Advancing 7T Perfusion Imaging by Pulsed arterial spin labeling: Using a parallel transmit coil for enhanced robustness and temporal SNR”.

2. ‘Perfusion’ labeled in the color bar of Figure 2 is not appropriate, which should be a normalized value such as ml/100g/min. It is better labeled as ‘Perfusion signal’ or ‘Perfusion weighted signal’. Besides, Figure 3 should also come with a color bar.

Response: We changed the label to ΔM, with ΔM defined as the subtraction of control and label images ($\Delta M=S_{control}-S_{label}$). We added this notion to the manuscript. For Fig 3, a color bar was added.

Changes:

FAIR perfusion-weighted maps were generated through pairwise subtraction of control and label images ($\Delta M=S_{control}-S_{label}$).

Figure changes:

Fig 2: Arterial spin labeling difference image (ΔM) from a single participant (P06), highlighting differences between the 2Tx and pTx systems. The pTx system generally shows more homogeneous signals (e.g. fewer right-left differences) than the 2Tx. The increased thickness of the slab-selective inversion (6cm, 9cm, and 12cm) resulted in an intensity reduction for both the 2Tx and pTx systems.

Fig 3: Single slice (#6) arterial spin labeling difference (ΔM) images from all 7 participants. An asymmetric right-left signal pattern can be seen on the 2Tx images for all slab thicknesses and participants. A more homogenous pattern is consistently observed in the pTx images. The contrast window is the same for all perfusion-weighted images.

**Reviewer #2:** This study investigates the effects of two transmit coils configuration on ASL perfusion imaging with FAIR using TR-FOCI for labeling and background suppression. The two configurations were: a) a parallel transmit system (pTX) with 32 Rx and 8 Tx channels, and b) a standard setup of 32Rx and 3Tx channels. This work showed that a pTx configuration is advantageous in ASL imaging at ultra-high magnetic fields given its less inhomogenous B1 field; however, CBF quantification is missing which would have given a more illustrative example of why it is better to use a pTx system. The two configurations were also tested in functional imaging. Both coils yielded robust fMRI measurements, and the tSNR was higher using the pTX system.

We thank the reviewer for all the positive feedback and valuable suggestions. In the second part of the first comment below we addressed the reviewer’s concern regarding the CBF quantification, and we have also addressed all other concerns raised by the reviewer.

Major Issues

1. To validate that a pTX setting was better than the standard system two quantitative measures were used: coefficient of variance (CoV) and temporal signal-to-noise ratio (SNR); however, as shown in equation two these two measurements are dependent from each other (or basically the same measurement i.e., CoV = 1/tSNR). CBF maps should be quantified in order to add robustness to the study.

**Response R2.C1.1 (metrics):**

The main assessment of the study was the temporal stability through CoV and tSNR, CoV was used to assess the temporal stability over the different slab inversion thicknesses, and the tSNR to assess the fMRI dataset. To further improve the robustness of the study, we now incorporated two additional metrics: signal-to-noise ratio (SNR) and contrast-to-noise ratio (CNR).

**Changes:**

Data analysis section

Additionally, signal-to-noise ratio (SNR) and contrast-to-noise ratio (CNR) were also assessed. The SNR was calculated using equation [3], and it was defined as the mean divided by the standard deviation of the gray-matter mask. The CNR was calculated using equation [4], μ_WM_ and μ_GM_ are the mean signal intensities of white matter and gray matter regions of interest, respectively. σ_GM_ and σ_WM_ are the standard deviation of the signal intensities in the gray matter and white matter regions of interest, respectively.

$SNR= \frac{\mu_{GM}}{\sigma_{GM}}$	[3]

$CNR= \frac{abs\left( \mu_{GM}-\mu_{WM} \right)}{\sqrt{\left( \sigma_{GM}^{2}+\sigma_{WM}^{2} \right)}}$	[4]

*Results section*

To assess the level of noise between the two coils, we investigated the SNR and CNR. For the SNR the left and the right gray matter ROIs were assessed. As can be seen in Fig 6 panel A, the median SNR was higher for pTx compared to the standard 2Tx coil in both ROIs and across the different slab inversion thicknesses. The SNR distribution across participants seems to be more spread in the 2Tx than in the pTx coil, specifically for the right GM region. Statistical assessment showed no significant differences between both coils. The CNR boxplot (Fig 6 panel B), displays the distribution of CNR across participants for both coils. We observed higher CNR for pTx than the 2Tx coil, and the paired t-test (Bonferroni corrected) showed a significant difference between both coil configurations (p<0.00001).

*Discussion section*

Additionally, we also evaluated the SNR across both ROIs and found a higher median for pTx compared to the 2Tx coil, however, this discrepancy lacked statistical significance. Interestingly, the SNR distribution across participants appears more widely dispersed with the 2Tx than the pTx coil, specifically for the right gray matter region. Given that our SNR measurements are likely driven by the signal intensities rather than the noise levels, we speculate that the observed variations have the same origin as the signal intensities that are observed between both hemispheres (see Figs 2&3). On the other hand, our dataset showed statistically significant differences in CNR between pTx and standard 2Tx coil. Higher CNR values indicate better differentiation between GM and WM regions, which can be crucial for lesion detection [2].

Fig 6: Assessing signal-to-noise ratio (SNR) (Panel A) and contrast-to-noise ratio (CNR) (Panel B) across different coil configurations. The boxplot illustrates the distribution of SNR and CNR values for the 2Tx and pTx coils. The pTx coil shows higher median SNR values (indicated by dark strokes) for both the left and right gray matter (GM), and these results are also consistent across slab inversion thicknesses. In terms of CNR, pTx yielded higher values than 2Tx. Statistical analysis using paired t-test (Bonferroni corrected) revealed a significant difference in CNR assessment for all thicknesses (p<0.00001), while no statistical difference was found in SNR. The level of significance is denoted by an asterisk symbol, with **** representing p<0.0001.

**Response R2C1.2 (CBF quantification):**

While we appreciate the reviewer’s suggestion regarding the quantification of CBF maps to enhance the robustness of our study, we respectfully disagree with this statement. Our study specifically focuses on comparing the performance of two distinct coils at 7T, by evaluating the differences between these coils in terms of image quality (tSNR, SNR, and CNR). By not quantifying CBF, we aim to highlight the potential differences in coil variations, which could be masked or normalized through quantification (i.e. make the difference insensitive to coil variations). Thus, while we acknowledge the importance of robust quantification in certain contexts, we believe that in the scope of our study, the omission of CBF quantification allows for a more direct comparison of coil performance, thereby providing valuable insights into the practical implications of using different coils at 7T.

**Changes:**

FAIR perfusion-weighted maps were generated through pairwise subtraction of control and label images ($\Delta M=S_{control}-S_{label}$). It is worth mentioning that while we acknowledge the importance of robust quantification in certain contexts, in the scope of this study, the omission of CBF quantification allows for a more direct comparison of coil performance. Hereby, we adhere to the principle of staying as close to the measured data as possible when analyzing the quality of the data.

2. Line 74: it would also allow for higher perfusion imaging resolution as seen in <https://doi.org/10.1371/journal.pone.0215998>

**Response R2.C2:**

We added the suggested reference.

**Changes:**

Moreover, it would also allow longer readout acquisitions, increased brain coverage, as well as higher spatial resolution perfusion images [3].

3. Line 152: I would show here in a figure the coils b1 maps or give a reference to the b1 maps showed in the supplementary material. Also, for this figure in the supplementary materials the scale and color bar are missing.

**Response R2.C3:**

The DREAM B1 map is the scanner-generated map. The maps are normalized. Thank you for pointing out the missing scale and color bar, which we now added. We also added a reference to the B1 maps in the supplementary material. Now referred to as S1 Fig.

S1 Fig

4. Was whole brain coverage achieved? If yes, it will be worth to show those images, or a comparison of maximum brain coverage achieved with both systems (pTx versus standard), as whole brain coverage is also specified in the introduction as one of the advantages of acquiring ASL at ultra high magnetic fields.

**Response R2.C4**:

While ultra-high magnetic field strengths do facilitate larger brain coverage, it is important to note that we did not explore whole brain coverage protocols in the present study. This was mainly driven by our focus on functional ASL applications for which a more focused FOV is acceptable, specifically when it would allow higher temporal resolution.

5. What correction method for multiple comparisons was used for the fMRI study?

**Response R2.C5:**

We did not conduct a voxel-wise multiple comparison analysis as part of the general linear model (GLM) fit. Instead, within FSL FEAT, we utilize a cluster threshold approach, with a specified z threshold equal to 3.1, as indicated in the manuscript. All the analyses were performed at the individual level and based on ROIs. To address multiple comparisons, we used Bonferroni correction for these assessments.

6. Line 233 : were t-test comparisons corrected for multiple comparisons?

**Response R2.C6**:

Yes, all t-tests were corrected for multiple comparisons with Bonferroni correction. We added this notion throughout the manuscript.

*Methods section:*

Changes: The statistical assessment was conducted in R version 4.2.1 [4], using t-tests with alternative hypotheses that the means (pTx vs 2Tx) are different. To address multiple comparisons, we applied Bonferroni correction.

*Results section:*

Changes: A paired t-test (Bonferroni corrected) revealed a significant difference in both hemispheres for the two labeling thicknesses, 6cm, and 9cm.

7. Line 240 in the caption of figure 4 caption: the wording of two different ROIs is confusing; these are the same right/left hemisphere gray matter masks, right?

**Response R2.C7**:

Thank you for pointing this out. We changed the caption accordingly.

**Changes:**

*Fig.4 Caption*

Coefficient of variance (CoV) vs. additional (label thickness) slab inversion thickness for right and left masks and coils (2Tx and pTx)…

8. Line 255 : was the tSNR significant higher for the pTX coil compared to the 2Tx coil?

**Response R2.C8:**

Text in line 255 refers to the fMRI dataset, and for this dataset, we did not find a statistical significance for the alternative hypothesis of tSNR being different for parallel transmit (pTx) compared to standard 2Tx (p-value=0.15). We explicitly stated this notion in the manuscript.

**Changes:**

… albeit, not statistically significant

9. Line 272: it might be worth to address in the discussion why do you think P04 displayed decreased z-values for pTX relative to 2Tx (something that was not really expected).

**Response R2.C9:**

We thank the reviewer for this question. While our processing pipeline did not identify issues of excessive motion, we acknowledged the possibility that the differences in the participant’s engagement with the task could have influenced the observed decreased z-values while using pTx coil.

**Changes:**

*Fig.9 Caption*

While our processing pipeline did not identify issues of excessive motion for participant P04, we acknowledge the possibility that the differences in the participant’s engagement with the task could have influenced the observed decreased z-values while using pTx.

10. Caption in Figure 1 of the supplementary material does not specify the preinversion thickness used for these FAIR acquisitions.

**Response R2.C10:**

Thank you for pointing this out. The maps shown are from the 6 cm slab inversion thickness.

**Changes**:

S2 Fig: Six slices (from 6-11) of perfusion-weighted ASL from all seven participants highlighting differences between the 2Tx and pTx systems. The pTx system generally shows a more homogeneous signal (e.g. fewer right-left differences) than the 2Tx. The perfusion maps are from the 6 cm slab inversion thickness.

Minor issues

1. Line 22: A in arterial spin labeling should not be capitalized. Overall, there is no consistency between capitalized and non-capitalized description of abbreviations, e.g., (line 27) flow-sensitive alternating inversion recovery (FAIR) or (line 28) Time-Resolved Frequency Offset corrected Inversion (TR-FOCI). Please choose a style and keep it constant throughout the manuscript.

**Response R2.C11:**

We appreciate the reviewer for pointing this to our attention. We have revised all descriptions of abbreviations to be in lowercase and have made changes throughout the manuscript. All the changes are highlighted in the revised manuscript.

2. Line 45: BOLD fMRI has not been previously described or spelled out.

**Response R2.C12:**

Description added to Introduction section, line 25.

3. References are missing in line 63 and 65.

**Response R2.C13:**

We added references accordingly.

4. Line 66: It is recommended not to use contractions in formal writing (i.e., ASL’s)

**Response R2.C14:**

We thank the reviewer for pointing this out. We have made appropriate changes to the manuscript.

**Changes:**

Scanning at a higher field strength not only holds the key to improving the intrinsic low SNR of ASL.

5. Line 145: C in Control should be in low case

**Response R2.C15:** We changed accordingly.

6. Line 170: G in Gray-matter should be in low case. These style errors are constant through-out the document please correct.

**Response R2.C16:** We have made the necessary adjustments to the manuscript.

7. Line 231: I think the sentence should read: …, meaning that there is more perfusion signal loss at larger inversion slab widths in the 2Tx compared to the pTx coil.

**Response R2.C17:** We have made the correct adjustments in the manuscript.

8. Line 298: add citation

**Response R2.C18:** A reference was included.

9. Line 302-308: very interesting

10. Line 317: capitalize ROIs

**Response R2.C20:** We have made the adjustments in the manuscript.

11. Line 319-320: UP definition is missing.

**Response R2.C21:**

Thank you for bringing this to our attention. ‘UPs’ stands for ‘universal pulses’, and we have incorporated this clarification into the Discussion section.

*Changes:*

… like Universal pulses (UPs) or kt-points…

**Reviewer #3:** This manuscript presents a pTx implementation of a pulsed arterial spin labeling technique at ultra-high field for brain perfusion imaging. They demonstrate a comparison of two coil system setups: 2 channel transmit and 8 channel transmit pTx. Two different experiments were performed to evaluate the coil performances. Authors claim the pTx system produces a more homogenous transmit field which translated into improved perfusion signal acquisition.

I agree that ASL can greatly benefit from ultra-high field strength. The use of pTx is becoming a popular approach to combat the challenges which are more evident at 7T. However, there are several technical problems and gaps in the proposed methodology which lack clarity and are of major concern. This leads to many assumptions which readers may need to make. It appears the manuscript focuses more on a coil comparison rather than the potential capabilities of pTx as implied in the title. It is unclear where the capabilities of using pTx are showcased beyond using the 8Tx pTx coil. I suggest the following concerns are addressed prior to recommendation of publication.

We thank the reviewer for the time taken to review the present manuscript. We appreciate all positive comments and suggestions. We understand the reviewer’s concern regarding the use of pTx capabilities. We specifically discuss this in the second response below and some of the follow-up comments.

Major comments:

1. There are various ASL labeling schemes which can be broadly categorized as spatially selective or velocity selective. Select statements made in the introduction regarding spatial coverage and dependence on arterial transit times is generally only applicable to spatially selective ASL schemes (PASL and pCASL). It will be insightful to elaborate and acknowledge in the introduction other ASL techniques which exist that are not spatially selective such as velocity selective ASL and their progress with using pTx.

**Response R3.C1:**

We thank the reviewer for pointing this out. While we appreciate the importance of velocity-selective labeling (VSL) techniques in the broader context of ASL methodologies, we want to emphasize that our manuscript primarily focuses on spatially-selective ASL techniques. This focus is now emphasized throughout the introduction. We acknowledge the existence of VSASL techniques in the text, mentioning a recent review. Additionally, we have referenced a recent study that explores the application of pTx pulses for excitation using VSASL. We became aware of this study when it was published after the submission of the manuscript.

**Changes:**

*Introduction:*

Line 30: It is worth pointing out that the present study is primarily focused on spatial selective techniques (and then especially pulsed ASL). The advantages and disadvantages of pTx might be different for velocity-selective labeling techniques, for specific information about velocity-selective labeling (VSASL) techniques, the reader is referred to [5].

Line 33: The fundamental concept of signal generation in spatially selective ASL is the manipulation of the longitudinal magnetization of water present ...

*Discussion:*

To date, the use of tailored pTx pulses such as kt-points and UPs in ASL literature is scarcer, especially for similar purposes as in BOLD fMRI studies, specifically targeting whole brain signal homogeneity. In principle, employing these methods in ASL fMRI could offer advantages such as increased tSNR, improved signal homogeneity, and potentially alleviating power deposition constraints. Initial insights demonstrated the benefits of these pulses in velocity-selective ASL [6].

2. Line 118 – In section 2.2 Data acquisition, it is briefly mentioned that B1-shimming was implemented over the entire brain. I understand separate volunteer data was used to achieve the B1-shim however there is ambiguity in the description. Please provide more detail on the size of volunteer data to calculate the shim parameters and whether this was a universal B1-shim applied for all subjects scanned for the study. Did you consider using a subject tailored shim or tailored pTx pulses for better B1 correction? If not, why? Please also specify in what aspect of the full ASL FAIR sequence was this shim applied to?

**Response R3.C2:**

We achieved a close-to-circularly polarized mode (CP mode) through B1-shimming across a cohort of 16 participants. Opting for a universal shimming method, rather than a subject-specific calibration, was motivated by the time-consuming nature and expertise required for the calibration. Our primary goal was to investigate whether the pTx coil could surpass the performance of the 2Tx coil in an application-relevant setting. Therefore, subject-tailored methods were not utilized. We added this notion in the manuscript including the new title. We also would like to clarify that the same shim was applied for labeling and imaging.

3. Line 120 – This next point follows on from comment 2. It is unclear where pTx capabilities is utilized despite the use of a pTx coil. The authors describe adiabatic tr-FOCI pulses are used for the labeling and two background suppression pulses. Adiabatic pulses are insensitive to B1 variation. They don’t require a pTx coil to be implemented effectively. My understanding is that these adiabatic pulse elements is what contributes significantly to the efficiency of the labeling and as a result the perfusion signal. This then only leaves the 2D-EPI excitation pulses and saturation modules. It is not mentioned and therefore unclear where exactly the pTx was applied to benefit the ASL technique evaluated.

**Response R3.C3:**

We thank the reviewer for pointing this out. We want to clarify that our study did not utilize parallel transmission (pTx) capabilities, such as tailored RF pulses or subject-specific shim. Our primary focus was on hardware comparison, and we have clarified this distinction in the manuscript.

**Changes related to R3.C2 &R3.C3:**

Line 107: No personalized RF-shim or other pTx pulses were used here to limit the comparison to the hardware used.

4. Line 152 – Was there any verification in the setup to ensure a fair comparison of the two coils? Authors mentioned a different maximum B1 limit was applied for the two coils respectively. There is a significant difference between the maximum B1 limit. This leads me to assume significantly lower/capped reference voltage was used for the 2Tx coil. Using a lower reference voltage may result in an overall lower flip angle distribution. This appeared to translate in the B1 maps shown in the supplementary material. Could you explain how experiments were performed to give a fair comparison. It would be ideal to consider a baseline calibration of the reference voltage prior to acquisition of perfusion protocols.

**Response R3.C4:**

Apart from the difference in maximum B1 (limited by the amplifiers), we used both systems with their standard settings to provide a comparison in which neither system was unduly limited and, hence, achieved a fair comparison for users opting for one or the other system. The labeling pulse attributes, including nominal flip angle and B1 amplitude, were identical for both coils.

**Changes:**

For the pTx system, a maximum B_1_^+^ value of 18μT was used for the excitation pulses, this is the default maximum for this system.

5. Line 291 – The authors state that labeling is dependent on the distal location selected. I agree that this has an effect as there are associated blood transition times and T1 relaxation effects. However, they conclude pTx is more efficient for inversion. My understanding is adiabatic pulses were used for inversion. When set up to satisfy adiabatic conditions they are insensitive to B1 variation. This leads me to question whether the tr-FOCI pulses were optimised and whether the restriction of maximum B1 limit influenced the overall inversion fidelity of the pulses?

**Response R3.C5:**

The pulse shape and the other attributes of the TR-FOCI pulse were set to the optimal settings as stated in the original publication [7]. Additionally, all the RF pulse attributes including nominal flip angle and B1 amplitude were the same for both coils. This implies that no imperfect inversion was happening for the 2Tx setup, probably mainly originating from the achievable max B1.

**Changes:**

A FAIR Arterial Spin Labeling sequence was implemented using TR-FOCI RF-pulses [11] with 13ms of pulse duration, nominal flip angle of 2576 degrees, and B1 = 15μT. We did not optimize the adiabatic inversion pulse, and all RF attributes, including nominal flip angle and B1 amplitude, for each setup separately, i.e., they were kept consistent across both coils.

6. It is not easily understandable what the cause of the asymmetry that occurs in your acquired 2Tx data. You refer to the B1 maps supplied in the supplementary material and suggest that the cause is not due to excitation distribution instead it is the RF profile. My understanding of “RF profile of the coils” is that it relates to transmit sensitivity which is the generated B1 distribution. It is unclear whether the right-side bias is caused by coil geometric sensitivities, transmit efficiency or the B1-shim applied. Can you explain what you mean by “RF profile of the coils” incase I have misunderstood.

**Response R3.C6:**

We apologize for the unclear phrasing. There is indeed little to no difference between the B1+ profiles of the coils at the level of the imaged slices (the excitation profile), but there may be and probably is a difference in the transmit B1 profile of the coils at the level of the tag, which results in imperfect labeling efficiency and thus an asymmetric perfusion signal.

We have re-written this sentence as follows:

… but rather by the different patterns of B1 for the two coils at the level of the labeling.

7. Compared to other regions of the brain, the B1 in the motor cortex at 7T is relatively adequate. This may be one contributing explanation why the fMRI results were comparable between the two coil setups. Did you consider investigating and comparing other areas (e.g. visual cortex or cerebellum) where B1 is less sufficient to showcase the benefits/diversity of pTx?

**Response R3.C7:**

Similarly to the previous response, the excitation B1+ may be comparable, but the labeling efficiency is not (necessarily). Given the significant differences observed in CNR and CoV (between coils) with the 12 cm slab inversion thickness experiment, it’s plausible that similar benefits could be seen while using pTx coil in the visual cortex. For the cerebellum, the labeling areas are likely below the transmit coil’s reach or at least in the less-homogenous areas.

Minor comments:

8. Line 77 – There may be more suitable references to include that demonstrates comparative implementation of different adiabatic pulses for PASL.

- Zimmer F, O'Brien K, Bollmann S, Pfeuffer J, Heberlein K, Barth M. Pulsed arterial spin labelling at ultra-high field with a B 1 (+) -optimised adiabatic labelling pulse. MAGMA. 2016 Jun;29(3):463-73. doi: 10.1007/s10334-016-0555-2. Epub 2016 Apr 15. PMID: 27084187.
- Wang K, Shao X, Yan L, Ma SJ, Jin J, Wang DJJ. Optimization of adiabatic pulses for pulsed arterial spin labeling at 7 tesla: Comparison with pseudo-continuous arterial spin labeling. Magn Reson Med. 2021 Jun;85(6):3227-3240. doi: 10.1002/mrm.28661.

**Response R3.C8:**

We thank the reviewer for the suggestion. We included these papers in our reference list.

9. Line 88 – Wording is confusing as two different concepts are trying to be conveyed here. SAR constraints are stringent at UHF whereas sequences can be less SAR intensive compared to others. PASL uses multiple adiabatic pulses which are SAR intensive. Could you please elaborate why you claim PASL may be less SAR intensive compared to other ASL techniques?

**Response R3.C9:**

Indeed, clarification is needed for the sentence in question. Firstly, we wanted to highlight that recent ASL-pTx studies predominantly focused on enhancing labeling efficiency by employing B1 shimming or implementing tailored pulse strategies, such as the VERSE pulse. These efforts were primarily aimed at improving the performance of pCASL sequences using pTx techniques.

Secondly, we aimed to emphasize that, despite the advancements in pCASL techniques, many studies still opt for pulsed ASL (PASL) sequences, especially in ultra-high field (UHF) experiments. The preference is often attributed to the lower SAR deposition of PASL sequences, as compared to pCASL. This is especially advantageous for ASL-fMRI, where background suppression pulses and the additional pulses to control bolus duration for the quantification of CBF maps are typically not used. In this context, the longer train of RF pulses which is used for tagging in pCASL significantly increases the SAR deposition. We have split the sentence to separate the two points.

**Changes:**

Previous studies have optimized and evaluated strategies such as B1-shimming or tailored RF pulses in ASL [8–10]. These investigations primarily aimed to enhance the labeling efficiency of pseudo-continuous ASL (pCASL) using pTx techniques. However, it is worth noting that, for functional experiments, less SAR-intensive PASL sequences are often preferred at UHF.

10. Figure 1 shows the coverage and location of the labeling and imaging regions. It would benefit to show a full sequence diagram with all sequence elements.

**Response R3.C10:**

We included the sequence diagram with all relevant elements. See Fig 1.

**Changes:**

Fig 1: Experimental design illustrating the Pulsed Arterial Spin Labeling (PASL) FAIR sequence. In panel (A) Experiment 1, the location of the control and labeling planes is depicted, followed by an example of possible additional slab inversion thicknesses. The imaging plane is represented in green, the non-selective inversion pulse (Control) in blue, the slab selective inversion pulse (labeling) in orange, and the coil coverage in red. In panel (B) Experiment 2, the protocol for the fMRI experiment is outlined along with the designed fMRI stimulus. The stimulus involved right-hand finger tapping every 33 seconds after the baseline (also 33 seconds), followed by a 33 rest condition (indicated by a cross sign). The total experiment time was 6 minutes and 5 seconds. Panel (C) shows the pulse sequence diagram with all relevant elements.

11. It may be insightful to detail the differences in coil configuration and geometry where appropriate since limited pTx pulse capabilities are implemented.

**Response R3.C11:**

We added the following details to the manuscript.

**Changes:**

We utilized two commercially available head coils in our study (Nova Medical Inc, Wilmington, USA). The first coil, known as 32Rx2Tx or 2Tx, is equipped with 32 receive phased array elements and two transmit channels. This coil features a circularly polarized transmit coil design with a helmet-shaped insert containing 32 receive elements. The second coil, referred to as pTx, incorporates eight transmit elements arranged cylindrically around the head, the 32-receive insert is organized as for 2Tx receive. The pTx coil employs a close-to-circularly polarized mode achieved through B1-shimming across the entire brain using data from a separate group of volunteers.

12. Line 299 – Could you please explain what you mean by “calibration of B1”.

**Response R3.C12:**

Calibration of B1 relates to the amount of power required for specific flip angles.

**Changes:**

… overall B1 amplitude

13. Line 302 – Wording implies there is a difference map calculated between the two independently acquired B1 maps of the two coils.

**Response R3.C13:**

We changed the text accordingly.

**Changes:**

different patterns of B1 for the two coils at the level of the tag.

14. Line 320 – There was initial insights demonstrated in the benefits of using tailored pTx pulses for velocity selective labelling at 7T which are directly applicable to ASL techniques.
- Wu C-Y, Jin J, Dixon C, Maillet D, Barth M, Cloos MA. Velocity selective spin labeling using parallel transmission. Magn Reson Med. 2024; 91(4): 1576-1585. doi: 10.1002/mrm.29955

**Response R3.C14:**

We would like to thank the reviewer for pointing out this reference which was published after submission of the current manuscript. We have incorporated it and changed the sentence as follows, please see R3.C1

**References**

1.	Teeuwisse WM, Webb AG, van Osch MJP. Arterial spin labeling at ultra-high field: All that glitters is not gold. Int J Imaging Syst Technol. 2010;20: 62–70. doi:10.1002/ima.20218

2.	Dury RJ, Falah Y, Gowland PA, Evangelou N, Bright MG, Francis ST. Ultra-high-field arterial spin labelling MRI for non-contrast assessment of cortical lesion perfusion in multiple sclerosis. Eur Radiol. 2019;29: 2027–2033. doi:10.1007/s00330-018-5707-5

3.	Mora Álvarez MG, Stobbe RW, Beaulieu C. High resolution continuous arterial spin labeling of human cerebral perfusion using a separate neck tagging RF coil. PLoS ONE. 2019;14: e0215998. doi:10.1371/journal.pone.0215998

4.	R Core Team. R: A Language and Environment for Statistical Computing. Vienna, Austria: R Foundation for Statistical Computing; 2022. Available: https://www.R-project.org/

5.	Qin Q, Alsop DC, Bolar DS, Hernandez-Garcia L, Meakin J, Liu D, et al. Velocity-selective arterial spin labeling perfusion MRI: A review of the state of the art and recommendations for clinical implementation. Magn Reson Med. 2022;88: 1528–1547. doi:10.1002/mrm.29371

6.	Wu C-Y, Jin J, Dixon C, Maillet D, Barth M, Cloos MA. Velocity selective spin labeling using parallel transmission. Magn Reson Med. 2024;91: 1576–1585. doi:10.1002/mrm.29955

7.	Hurley AC, Al-Radaideh A, Bai L, Aickelin U, Coxon R, Glover P, et al. Tailored RF pulse for magnetization inversion at ultrahigh field. Magn Reson Med. 2010;63: 51–58. doi:10.1002/mrm.22167

8.	Tong Y, Jezzard P, Okell TW, Clarke WT. Improving PCASL at ultra-high field using a VERSE-guided parallel transmission strategy. Magn Reson Med. 2020;84: 777–786. doi:10.1002/mrm.28173

9.	Meixner CR, Eisen CK, Schmitter S, Müller M, Herrler J, Hensel B, et al. Hybrid -shimming and gradient adaptions for improved pseudo-continuous arterial spin labeling at 7 Tesla. Magn Reson Med. 2022;87: 207–219. doi:10.1002/mrm.28982

10.	Wang K, Shao X, Yan L, Ma SJ, Jin J, Wang DJJ. Optimization of adiabatic pulses for pulsed arterial spin labeling at 7 tesla: Comparison with pseudo-continuous arterial spin labeling. Magn Reson Med. 2021;85: 3227–3240. doi:10.1002/mrm.28661
