# Supplementary material for: Advancing 7T perfusion imaging by pulsed arterial spin labeling: Using a parallel transmit coil for enhanced labeling robustness and temporal SNR
Source: PLoS One. 2024 Aug 26;19(8):e0309204. doi: 10.1371/journal.pone.0309204 (PMC11346640; doi:10.1371/journal.pone.0309204)
Supplement: Attachment — Submitted filename: response_reviewers_final.docx [file pone.0309204.s003.docx]

**Journal requirements**

We have updated the list of references in the manuscript.

**Response to reviewers**

Reviewer #1: Thank you for revising your manuscript and including the requested information. My previous concerns were all well addressed but there are a few more comments for the added contents (The following line numbers refer to the manuscript with tracked changes).

1. Line 110: ‘A FAIR Arterial Spin Labeling sequence was implemented using TR-FOCI RF-pulses [25] with 13ms of pulse duration, nominal flip angle of 2576 degrees, and B1 = 15μT’; Line 143: ‘For the pTx system, a maximum B1+ value of 18μT was used for the excitation pulses’; Line 144: ‘For the 2Tx, a maximum B1 of 12μT was used for the excitation pulses to accommodate amplifier limitations’. Is there a specific reason why the excitation pulses used different B1+ values for the two coils while TR-FOCI RF-pulses used another but the same maximum B1+ value?

Response: We thank the reviewer for the positive comments and suggestions. RF power is chosen based on the ability to run the sequence with a particular setting, with power deposition (SAR), transmit coil and amplifier properties being the typical constraints. When a potential SAR or power limit is encountered, the amplitude of the excitation pulses is reduced, keeping the amplitude of contrast-generating pulses, like inversion pulses constant. In our case, the ASL-FAIR has numerous RF pulses that can significantly increase the power deposition. Additionally, the pTx coil achieves decreased SAR, allowing for higher maximum B1+ values compared to the 2Tx coil.

2. Line 186: ‘μGM and μWM are the mean signal intensities of white matter and gray matter regions of interest, respectively’ should be ‘of gray matter and white matter regions of interest, respectively.

Changed: “mean signal intensities of gray matter and white matter regions of interest, respectively.”

3. How were the white matter masks generated?

Response: We created the white matter binary masks utilizing the white matter tissue map generated through the SPM12 segmentation.

Changes: “To create the gray-matter and white-matter masks, we used the segmentation tool from SPM12 on the averaged perfusion-weighted images. Gray and white matter binary masks for the left and right hemispheres were created manually using iTK-SNAP by editing the segmented gray-matter and white-matter masks.”

4. Line 197 Equation 4: the CNR is typically defined as abs(S_GM-S_WM)/σ. Here the author tried to use the noise information from both the grey matter and the white matter. I expect the denominator to be sqrt((σ_GM^2+ σ_WM^2)/2) rather than be sqrt(σ_GM^2+ σ_WM^2). Nevertheless, this is only a scaling and will not affect the outcomes.

Response: We agree with the reviewer’s comment. The CNR equation we used was based on the extension developed by the MRIQC pipeline (Esteban et al., 2017) from fMRIprep. We have added this notion to the manuscript.

Changes: “Both SNR and CNR equations are derived from the publicly available quality assurance pipeline (MRIQC).”.

5. Figure 6B: why were the right and left hemispheres not differentiated for the CNR boxplots?

Response: We have updated Figure 6B to differentiate the right and left hemispheres. As shown in the updated figure below, the results were very similar when separated by right and left hemispheres.

Fig 6: Assessing signal-to-noise ratio (SNR) (Panel A) and contrast-to-noise ratio (CNR) (Panel B) across different coil configurations. The boxplot illustrates the distribution of SNR and CNR values for the 2Tx and pTx coils. The pTx coil shows higher median SNR values (indicated by dark strokes) for both the left and right gray matter (GM), and these results are also consistent across slab inversion thicknesses. In terms of CNR, pTx yielded higher values than 2Tx. Statistical analysis using paired t-test (Bonferroni corrected) revealed a significant difference in CNR assessment for all thicknesses (p<0.00001) for both the right and left hemispheres, while no statistical difference was found in SNR. The level of significance is denoted by an asterisk symbol, with **** representing p<0.0001.

Reviewer #2: I appreciate the authors addressing my previous comments. This study investigates the effects of two transmit coils configuration on ASL perfusion imaging with FAIR using TR-FOCI for labeling and background suppression. The two configurations were: a) a parallel transmit system (pTX) with 32 Rx and 8 Tx channels, and b) a standard setup of 32Rx and 2Tx channels. This work showed that a pTx configuration is advantageous in ASL imaging at ultra-high magnetic fields given its less inhomogeneous B1 transmit field. Additional, CoV, CNR and SNR measurements were assessed to add robustness to the study. The two configurations were also tested in functional imaging. Both coils yielded robust fMRI measurements, but the tSNR was higher using the pTX system.

Major Issues:

1. I think it is important to clarify why SNR was not calculated using the standard deviation of noise in the background (air). Presumably, this is why no statistically significant difference was seen in the SNR assessment between the two coils. I know the use of parallel imaging and transmit coils make this measurement complex, but it will be worth to comment on that.

Response: We thank the reviewer for the positive comments and suggestions. Using parallel imaging can influence the statistical and spatial distribution of noise. The noise distribution in parallel imaging is described by the spatially varying geometry factor (g-factor) and depends on parameters such as the coil geometry, phase encoding direction, and acceleration factor (Dietrich et al., 2007), and the calculation of the SNR using the same ROIs is commonly suggested as an alternative approach (Dietrich et al., 2007), the approach used in the present study was based on the quality assurance pipeline (MRIQC) (Esteban et al., 2017), which is also part of the fMRIprep quality check pipeline. We added this notion to the manuscript.

Changes: The SNR equation using the same ROI is based on a previous recommendation (Dietrich et al., 2007) to minimize the influence of differences in the spatial distribution of the noise in parallel imaging acquisition. Both SNR and CNR equations are derived from the publicly available quality assurance pipeline (MRIQC) (Esteban et al., 2017).

Minor issues:

1. Line 49: This sentence is a phrase and not a complete sentence, it should be connected to the next sentence with a conjunction, in this case using not only…, but also… e.g., Scanning at a higher field strength not only holds the key to improving the intrinsic low SNR of ASL, but also recent research has...

Response: We have made the suggested changes accordingly.

Changes: “Scanning at a higher field strength might hold the key to improving the intrinsic low SNR of ASL as recent research has revealed that a higher static magnetic field (B0) leads to a supralinear improvement in SNR of a traditional MRI scan [10], which would also enhance the sensitivity and precision of ASL measurements.”

2. Line 341: The should be a semicolon before however instead of a comma, as it is being used as a conjunction.

Response: We have made the suggested changes accordingly.

Changes: “Additionally, we also evaluated the SNR across both ROIs and found a higher median for pTx compared to the 2Tx coil; however, …”.

3. Line 369: I think the flow of the paragraph will be improved if this sentence is start as.. Our fMRI experiment .. since you are previously describing other studies it can be a bit confusing when reading it at a first glance as The fMRI experiment…

Response: Thank you, we have made the suggested changes accordingly.

Changes: “Our fMRI experiment consistently elicited …”

Reviewer #3: Thank you for the corrections that you have made in this revision. The authors have mostly addressed my comments satisfactorily.

1. (R3.C5) There is still a concern on clarity with regards to the achieved B1+=15uT for the TR-FOCI RF pulses. The authors clarified that the pulse attributes were kept consistent across both coils. However, there is a discrepancy in the manuscript (line 144) which states, “For the 2Tx, a maximum B1+ of 12uT was used for the excitation pulses to accommodate amplifier limitations”. Given this amplifier limitation, how is the B1+=15uT achieved on the 2Tx?

Response: We thank the reviewer for the positive comments and suggestions. For the system used (7T Philips Achieva), when SAR limitations are encountered, changes in B1 maximum amplitude are applied only to the excitation pulses. Inversion pulse attributes, such as B1, pulse length, and flip angle are kept as predefined. Of course, the inversion pulses do not exceed safety limits. Please also check response R1.C1. We have added this notion to the manuscript.

Changes: For the pTx system, a maximum B1^+^ value of 18 μT was used for the excitation pulses; this is the default maximum for this system. For the 2Tx, a maximum B1+ of 12 μT was used for the excitation pulses to accommodate amplifier limitations. For the system used in the present study, changes in the B1 maximum are applied only to the excitation pulses. The inversion pulse attributes, such as B1 amplitude, pulse length, and flip angle were kept as predefined. The inversion pulses do not exceed safety limits. Apart from the excitation B1^+^ value, all sequence parameters were kept the same for all participants.

2. (R3.C6) Is it possible to show multiple slices of the B1 maps (S Fig. 1) which covers the labeling regions highlighted in Figure 1 to verify the claims of potential differences of the B1+ profile of the coil at the level of the tag?

Response: We have updated the Supplementary Figure 1 (S Fig. 1).

Changes:

S Fig 1: Representative B1^+^ maps (DREAM B1) from an individual participant. Panel A depicts the B1^+^ maps in sagittal, coronal and axial planes. Panel B shows the location of four slices, corresponding to the typical location of the labeling plane. Noticeably higher B1 values are observed in the pTx compared to the 2Tx B1 map. Panel C displays the location of the four slices.

Finally, there are some minor inconsistencies with abbreviations (e.g. B1+ vs B1) used throughout the manuscript. Otherwise, I’m quite satisfied with the authors’ response.

Response: We thank the reviewer for pointing this out. We have made the suggested adjustments in the manuscript.

**References**

Dietrich, O., Raya, J.G., Reeder, S.B., Reiser, M.F., Schoenberg, S.O., 2007. Measurement of signal-to-noise ratios in MR images: Influence of multichannel coils, parallel imaging, and reconstruction filters. J. Magn. Reson. Imaging 26, 375–385. https://doi.org/10.1002/jmri.20969

Esteban, O., Birman, D., Schaer, M., Koyejo, O.O., Poldrack, R.A., Gorgolewski, K.J., 2017. MRIQC: Advancing the automatic prediction of image quality in MRI from unseen sites. PLOS ONE 12, e0184661. https://doi.org/10.1371/journal.pone.0184661
